# Monocyte human leukocyte antigen-DR but not β-d-glucan may help early diagnosing invasive Candida infection in critically ill patients

**DOI:** 10.1186/s13613-021-00918-1

**Published:** 2021-08-21

**Authors:** Boris Jung, Clément Le Bihan, Pierre Portales, Nathalie Bourgeois, Thierry Vincent, Laurence Lachaud, Gerald Chanques, Matthieu Conseil, Philippe Corne, Pablo Massanet, Jean François Timsit, Samir Jaber

**Affiliations:** 1 Medical Intensive Care Unit, Montpellier University and Montpellier University Health Care Center, 34290 Montpellier, France; 2 PhyMedExp Laboratory, Montpellier University, INSERM, CNRS, CHRU Montpellier, 34295 Montpellier, France; 3 Département des Maladies Infectieuses et Tropicales, Montpellier University and Montpellier University Health Care Center, 34295 Montpellier, France; 4 Immunology Department, Montpellier University and Montpellier University Health Care Center, 34295 Montpellier, France; 5 Département de Parasitologie-Mycologie, Montpellier University and Montpellier University Health Care Center, UMR Mivegec, 34295 Montpellier, France; 6 Saint Eloi Department of Anesthesiology and Critical Care Medicine, Montpellier University and Montpellier University Health Care Center, 34295 Montpellier, France; 7 Department of Anesthesiology and Critical Care Medicine, Centre Hospitalier Universitaire Nîmes, 30000 Nîmes, France; 8 APHP Hôpital Bichat-Claude Bernard, Paris-Diderot University, 75000 Paris, France

**Keywords:** Beta D-glucan, mHLA-DR, Candidiasis, Septic shock

## Abstract

**Background:**

Precision medicine risk stratification is desperately needed to both avoid systemic antifungals treatment delay and over prescription in the critically ill with risk factors. The aim of the present study was to explore the combination of host immunoparalysis biomarker (monocyte human leukocyte antigen-DR expression (mHLA-DR)) and Candida sp wall biomarker β-d-glucan in risk stratifying patients for secondary invasive Candida infection (IC).

**Methods:**

Prospective observational study. Two intensive care units (ICU). All consecutive non-immunocompromised septic shock patients. Serial blood samples (*n* = 286) were collected at day 0, 2 and 7 and mHLA-DR and β-d-glucan were then retrospectively assayed after discharge. Secondary invasive Candida sp infection occurrence was then followed at clinicians’ discretion.

**Results:**

Fifty patients were included, 42 (84%) had a Candida score equal or greater than 3 and 10 patients developed a secondary invasive Candida sp infection. ICU admission mHLA-DR expression and β-d-glucan (BDG) failed to predict secondary invasive Candida sp infection. Time-dependent cause-specific hazard ratio of IC was 6.56 [1.24–34.61] for mHLA-DR < 5000 Ab/c and 5.25 [0.47–58.9] for BDG > 350 pg/mL. Predictive negative value of mHLA-DR > 5000 Ab/c and BDG > 350 pg/mL combination at day 7 was 81% [95% CI 70–92].

**Conclusions:**

This study suggests that mHLA-DR may help predicting IC in high-risk patients with septic shock. The added value of BDG and other fungal tests should be regarded according to the host immune function markers.

## Background

A keystone of sepsis is its association with systemic inflammation and immune-mediated damage [1]. However, it has been reported that along with systemic inflammation comes a simultaneous anti-inflammatory response, mostly represented by a decrease in monocyte human leukocyte antigen-DR (mHLA-DR) expression, T cell exhaustion and impaired ability of proinflammatory response all of them leading to post-aggression host immunoparalysis [2, 3], a condition associated with both hospital acquired infections and mortality [4]. The majority of hospital acquired infections are represented by bacterial infections, but a proportion of the critically ill patients will develop hospital acquired invasive Candida infection (IC). IC incidence is increasing nowadays [5] and is associated with an ICU mortality rate up to 50% [5–8]. Current pathogen-associated tools to help diagnose IC unfortunately either lack of sensitivity (e.g. cultures from sterile sites) or of specificity (clinical prediction scores, molecular biology, β-d-glucan wall biomarker detection) especially in the critically ill population [6, 9, 10]. The major diagnostic benefit of the recent β-d-glucan might be to help rule out IC as its negative predictive value has been reported to be about 90% [11]. Because delaying antifungal therapy initiation is a major determinant of clinical outcome [12, 13], intensivists desperately need better tools and/or bundles in the era of precision medicine to better screen patients at risk so their outcome can be improved antifungals over prescription being avoided [14].

The aim of the present study was to assess whether the combination of a host immunoparalysis biomarker (mHLA-DR) and a pathogen-associated biomarker (β-d-glucan) would help stratifying critically ill patients with septic shock at risk to develop secondary IC.

## Methods

### Study setting and patients

This prospective observational study (ClinicalTrials.gov identifier: NCT03136081) was performed in two adult ICUs of a University hospital from June 2014 to May 2015. The study was approved by the ethic committee (Comité de Protection des Personnes Sud-Mediterranée III, 2014-A00500-47) and followed the STARD guidelines [15]. In accordance with French law, informed consent was obtained by the patient or his/her next of kin. Were included adult patients admitted to the 2 participating ICUs with non-fungal septic shock or adult patients who developed septic shock during the ICU stay taking into account logistical issues (research support to optimize patients screening, preanalytic issues during the week ends, assays availability, restricted funding). Non-inclusion criteria were neutropenia (defined as a total leukocytes count < 500/mm^3^), immunosuppressive therapy, cancer-related chemotherapy in the last year, history of bone marrow or solid-organ transplantation. The patients were then split into two groups (IC and No-IC, NIC) according to the occurrence of IC during the ICU stay.

### Definitions

Septic shock was defined by evidence of infection and a systemic response to infection, in addition to a systolic blood pressure of < 90 mmHg, despite adequate fluid replacement, and a need for vasopressors for at least 1 h, according to the American College of Chest Physicians/Society of Critical Care Medicine Consensus Conference Committee criteria [16]. The diagnosis of IC was made based on the revised consensus definitions of invasive fungal infections developed by the European Organization for Research and Treatment of Cancer/Invasive Fungal Infections Cooperative Group and the National Institute of Allergy and Infectious Disease Mycoses Study Group [17]. IC was defined by culture positives for Candida spp. from blood, per-operative peritoneal fluid or another sterile site. NIC was defined by the absence of proved IC. The IC follow-up was not standardized and let at the discretion of the clinicians.

### Baseline assessment and clinical data collection

Clinical data were recorded at ICU admission: demographic characteristics, severity of underlying medical condition according to Simplified Acute Physiology Score II (SAPS II) [18], Sepsis-related Organ Failure Assessment (SOFA) score [19], the presence of comorbidities, reason for admission to the ICU and cause of septic shock. During the ICU stay, the following data were collected at days 0, 2 and 7; SOFA score, length of stay, duration of mechanical ventilation, need for vasopressor and survival at ICU discharge. Antibacterial and antifungal drugs were given according to the recommendation applied in a same way in both ICUs [20].

### Immunological data

EDTA-anti-coagulated tubes were collected on each visit day (days 0, 2 and 7) for blood cell count, lymphocyte phenotype, CD4 T-cell, CD8 T-cell count and mHLA-DR. Circulating mHLA-DR expression was assessed by flow cytometry (NAVIOS®; Beckman-Coulter) in accordance with the standardized recommendation [21, 22]. Monocytes were characterized on the basis of their CD14 expression. Results were expressed as the number of anti-HLA-DR antibodies per cell (AB/c). Clinicians were blinded for the immunological data results.

### Mycological data

Candida colonization was screened at days 0 and 7 in at least 3 sites from urine, gastric aspiration, tracheal aspiration and cutaneous swab. Candida Score [23] was calculated for each visit day. Colonization was defined when one nonsterile sample site was positive for Candida sp. Culture positive for IV catheter without positive blood culture was deemed to be a colonization. Multifocal colonization was defined when more than one non sterile sample site was positive for Candida sp. Standardized procedures for in vitro identification of microorganism were used according to the usual procedures of the local mycology laboratory. Clinicians were blinded for the mycological data results.

### Mycological biomarkers

Β-d-Glucan (BDG) was obtained on days 0, 2 and 7. Blood samples (15 mL) were centrifuged, separated into aliquots, and stored at − 80 °C until analysis performed at the end of the study. The BDG assay (Fungitell®, Associates of Cap Cod Inc., Easy Falmouth, MA, USA) was performed according to the manufacturer’s recommendations. The cutoff value was set according to the company recommendations at 80 pg/mL [24, 25]. None of the results was available to the physician in charge.

### Endpoints

The primary endpoint was the comparison of the kinetics during the first week after septic shock of mHLA-DR alone and in combination with BDG in IC and NIC patients.

### Statistical analysis

Data are expressed as mean ± SD or SEM for normally distributed data, and median with 95% confidence index (95%CI) for non-normally distributed data. Comparisons and biomarkers kinetics between IC and NIC patients were made at day 0, 2 and 7. Continuous variables were compared using Student’s t test for normally distributed variables and the Mann–Whitney rank-sum test for non-normally distributed variables. The Chi-square test or the Fisher exact test was used to compare categorical variables.

A cause-specific hazard model was built to assess the association of candida-related and immunologicals variables on the probability of death in the ICU or invasive Candida infection [26]. In this model, the occurrence of IC was the variable of interest, while death was considered as a competing event. Being discharged alive from the ICU was considered as a censored variable. Considering the low occurrence of candida infection only univariate analysis was performed. Candida variables and impaired immune function variables were fitted as time-dependent covariables. The direct effect on the risk of IC was estimated by cause-specific hazard ratio (csHR).

Analysis of sensitivity, specificity, positive predictive value and negative predictive value was calculated for mycological and immunological markers separately and in association. For each marker, value over/under the threshold was only considered if before an event (IC, death or ICU discharge). Delay of infection was estimated by the Kaplan–Meier method and compared between high- and low-risk patients (according to mHLA-DR and BDG values) with the log-rank test. A Monte Carlo simulation (considering the covariance between the two slopes) was also performed in order to assess the potentially best ROC curve. Expecting a 20% incidence of Candida sp positive samples following septic shock, a drop of 50% in mHLA-DR in the IC patients by day 7 and no significant difference between day 0 and day 7 in NIC patients, we estimated that 50 patients with septic shock would be needed assuming a bilateral test, an alpha risk of 0.05 and a power of 0.8. A P value of less than 0.05 was taken as the significance level. We used SAS 9.4 (Sas Institute, NC, USA), R 3.0.2 (Vienna, Austria) and GraphPad Prism 6 for all the statistical analyses.

## Results

**Fig. 1:**
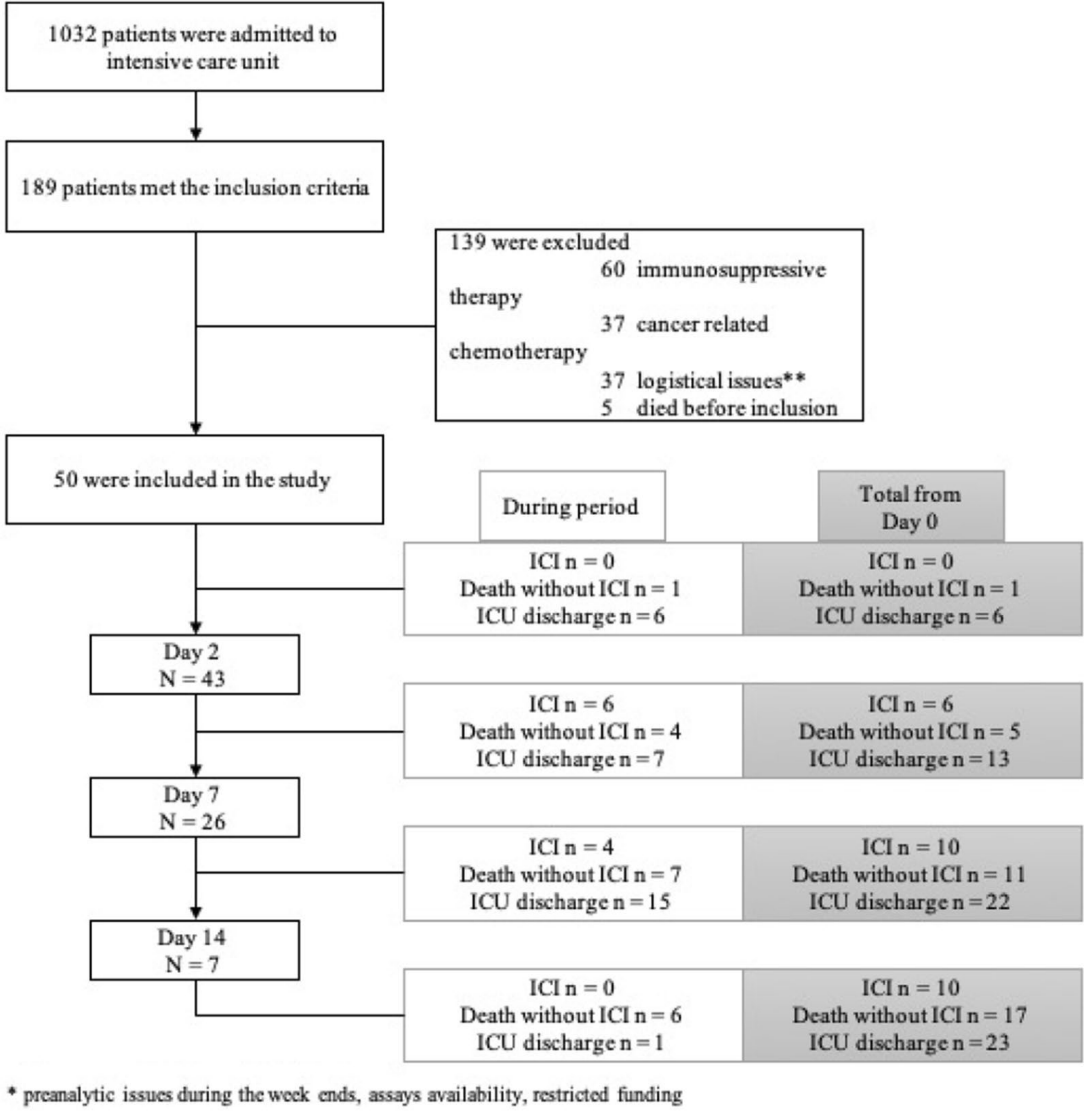
Flowchart. *IC* Invasive Candida Infection, *ICU* intensive care unit

Because of pre-analytic logistical issues, funding availability to facilitate screening and enrollment and logistical limitations in the assays availability, 50 patients met the inclusion criteria and were enrolled (Fig. 1). The baseline characteristics and outcome are shown in Table 1.

**Table 1:** Characteristics and outcome of the study population

	Overall population (*n* = 50)	No invasive candida infection (*n* = 40)	Invasive Candida infection (*n* = 10)	*p*-value
Age	66.4 [60.5–71.3]	66 [57.8–70.3]	69.5 [60.7–74.5]	0.343
Male	34 (68)	27 (67.5)	7 (70)	0.880
SAPSII at admission	50 [43–62]	48 [42–58]	58 [47–66]	0.143
SOFA score at admission	10 [7–12]	9 [7–12]	11.5 [7–13]	0.528
Past medical history				
Diabetes	15 (30)	12 (30)	3 (30)	1.000
NYHA III–IV heart insufficiency	8 (16.3)	8 (20.5)	0 (0)	0.117
Chronic renal failure	5 (10)	4 (10)	1 (10)	1.000
Cirrhosis	15 (30)	13 (32.5)	2 (20)	0.440
COPD	12 (24)	11 (27.5)	1 (10)	0.246
Cancer	14 (28)	7 (17.5)	7 (70)	0.001
Site of initial bacterial infection				
Community-acquired pneumonia	5 (10)	5 (12.5)	0 (0)	0.239
Health-care associated pneumonia	6 (12)	4 (10)	2 (20)	0.384
Intra-abdominal infection	17 (34)	11 (27.5)	6 (60)	0.052
Biliary tract infection	5 (10)	4 (10)	1 (10)	1.000
Urinary tract infection	2 (4)	2 (5)	0 (0)	0.470
Other infections	9 (18)	8 (20)	1 (10)	0.915
Risk factors for Candida infection at inclusion				
Candida colonization	34 (68)	27 (67.5)	7 (70)	0.880
Multifocal Candida colonization	32 (64)	24 (60)	8 (80)	0.239
Candida score	4 [3, 4]	3 [3, 4]	4 [4, 5]	0.071
Parenteral nutrition	20 (40)	15 (37.5)	5 (50)	0.470
Surgery	27 (54)	19 (47.5)	8 (80)	0.065
Abdominal surgery	23 (46)	15 (37.5)	8 (80)	0.016
Broad-spectrum antibiotherapy	50 (100)	40 (100)	10 (100)	1
Immunologicals and mycological parameters at inclusion				
Total lymphocytes	613 [349–992]	620 [391–1000]	546 [289–731]	0.434
CD8+T cells	73 [51–184]	71 [47–167]	124 [59–209]	0.343
CD4+T cells	279 [134–409]	279 [146–467]	245 [93–373]	0.493
NK cells	64 [29–106]	65 [30–118]	42 [29–66]	0.331
mHLA-DR	9477 [3605–15513]	9426 [3978–15668]	12,387 [3592–13180]	0.888
Beta-d-glucan	73 [22–145]	76 [16–181]	62 [34–107]	0.872
Outcome and treatments				
Antifungal therapy at inclusion	20 (40)	11 (27.5)	9 (90)	< 0.001
Mechanical ventilation, days	3 [1–10]	3 [1–6.5]	9 [2–22]	0.067
Vasopressors, days	2.5 [2–6]	2.5 [2–4.5]	4 [2–8]	0.458
ICU length of stay	7 [4–14.5]	7 [4–14]	9.5 [3–22]	0.596
Day in ICU before inclusion	2 [1, 2]	2 [1, 2]	2 [2–2]	0.616
Death	17 (34)	12 (30)	5 (50)	0.232

Categorical data are expressed as number and percentage. Continuous data are expressed as median and quartiles. Comparisons were made between patients with invasive Candida infection and without invasive Candida infection

*SAPS* Simplified Acute Physiology Score, *SOFA* Sequential Organ Failure Assessment, *NYHA* New York Heart Association, *COPD* chronic obstructive pulmonary disease, *mHLA-DR* monocytic human leucocyte antigen DR, *ICU* intensive care unit

Forty-three patients were followed until day 2, 26 patients until day 7. Ten patients developed an IC during follow-up. The median time from septic shock to IC was 11 days (IQR 4.25–12.25). A cancer was present in 14/50 patients (28%) and was more frequent in IC compared to NIC patients (70% vs. 17.5%, *p* = 0.001). There was no statistical difference in other baseline characteristics, ICU length of stay, mechanical ventilation and overall mortality between IC and NIC.

Peritonitis (22/50, 44%) and pneumonia (11/50, 22%) were the main sites of initial sepsis. Intra-abdominal candidiasis was the more frequent IC (*n* = 5), followed by candidemia (*n* = 4) and pleural infection (*n* = 1). A Candida Score ≥ 3 was found in all IC patients before infection occurred and in 32 (80%) NIC patients (*p* = 0.12). Antifungal therapy was used in 20 patients (40%). Preemptive treatment was administered in 11 (27%) NIC patients. Among the nine IC-treated patients, 5 (55%) received initial preemptive therapy that was pursued once the IC was confirmed while 4 (45%) received initial curative treatment. In one patient, the diagnosis of IC was made after ICU discharge and the treatment was not started. Echinocandins was the first agent in 45% (9/20) and fluconazole in 55% (11/20).

**Fig. 2:**
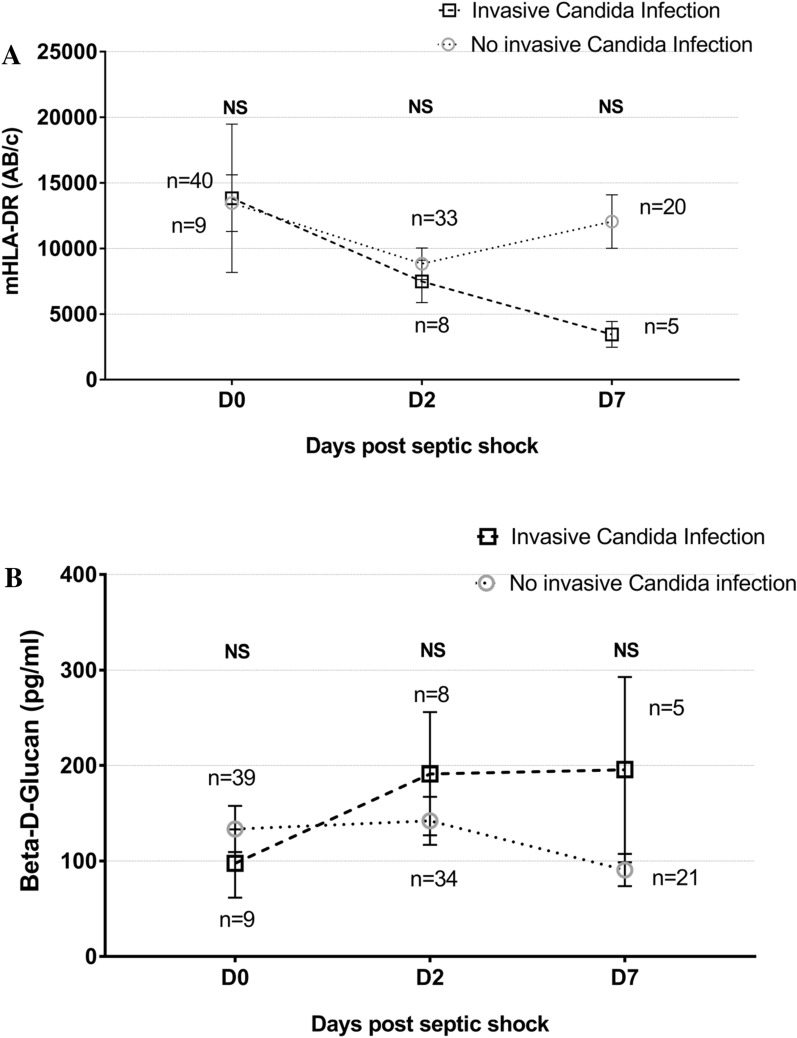
**A** Time course of mHLA-DR from day 0 to day 7 post septic shock. Results are expressed as mean ± SEM. Difference between IC and NIC from day 0 to day 7 is not significant. However, considering day 7 and day 0, mHLA-DR was different between IC and NIC patients. **B** Time course of β-d-glucan from day 0 to day 7 post septic shock. Results are expressed as mean ± SEM. Difference between IC and NIC is not significant

A total of 286 immunological and mycological blood samples were collected. At day 0 and at day 2, mean mHLA-DR expression was not different between IC and NIC patients and above the threshold of 8000 AB/c. At day 7, IC patients had a lower mHLA-DR expression than NIC patients (3451 ± 978 AB/c vs. 12,049 ± 2044 AB/c; p = 0.002) (Fig. 2A). At day 0, mean BDG was 97.5 ± 35.6 pg/mL and 133.5 ± 24.5 pg/mL for IC and NIC patients, respectively (p = 0.38) (Fig. 2B).

In the cause-specific univariate model (Table 2), the time-dependent value of mHLA-DR < 8000 Ab/c was not significantly associated with IC (cs-HR 6.83 [0.81–57.53]). An mHLA-DR < 5000 Ab/c was associated with IC (cs-HR of 6.56 [1.24–34.61]) but not significantly associated with death (cs-HR 2.15 [0.43–10.68]).

**Table 2:** Univariate cause-specific hazard ratio of invasive candida infection (competing risks: death, censored: alive discharge from ICU)

	Cause-specific HR of invasive Candida infection		Cause-specific HR of death	
	Cs-HR	95%CI	Cs-HR	95%CI
Past history of cancer	7.17	[1.38–37.36]	1.38	[0.25–7.61]
SAPS II (time-dependent)	1.06	[1.01–1.11]	1.01	[0.96–1.07]
SOFA score (time-dependent)	1.33	[1.08–1.62]	1.5	[1.06–2.13]
Immunological markers (time-dependent)				
Total lymphocytes < 1100/mm^3^	1.24	[0.15–10.29]	1.04	[0.12–8.96]
Total lymphocytes < 500/mm^3^	3.83	[0.74–19.81]	1.49	[0.3–7.4]
CD4+ T cells < 200/mm^3^	4.49	[0.87–23.23]	3.6	[0.66–19.67]
CD8+ T cells < 80/mm^3^	0.74	[0.16–3.31]	6.56	[0.79–54.55]
NK cells < 60/mm^3^	1.24	[0.23–6.7]	0.52	[0.11–2.44]
mHLA-DR < 5000 Ab/cells	6.56	[1.24–34.61]	2.15	[0.43–10.68]
mHLA-DR < 8000 Ab/cells	6.83	[0.81–57.53]	4.42	[0.52–37.95]
Fungals markers (time-dependent)				
Empiric antifungal therapy	1.15	[0.25–5.22]	0.31	[0.04–2.56]
Beta-d-glucan > 80 pg/mL	0.63	[0.14–2.86]	2.07	[0.4–10.77]
Candida score (total score)	2.09	[0.74–5.92]	0.43	[0.19–0.96]
Number of Candida colonization sites	1.17	[0.59–2.32]	1.52	[0.68–3.37]
Colonization index > 0.5	4.1	[0.38–44.5]	2.91	[0.23–36.23]

Evaluation of time-dependent mycological and immunological markers after septic shock and inclusion (from day 2 to day 14)

*SAPS* Simplified Acute Physiology Score, *SOFA* Sequential Organ Failure Assessment, *mHLA-DR* monocytic human leucocyte antigen DR

**Fig. 3:**
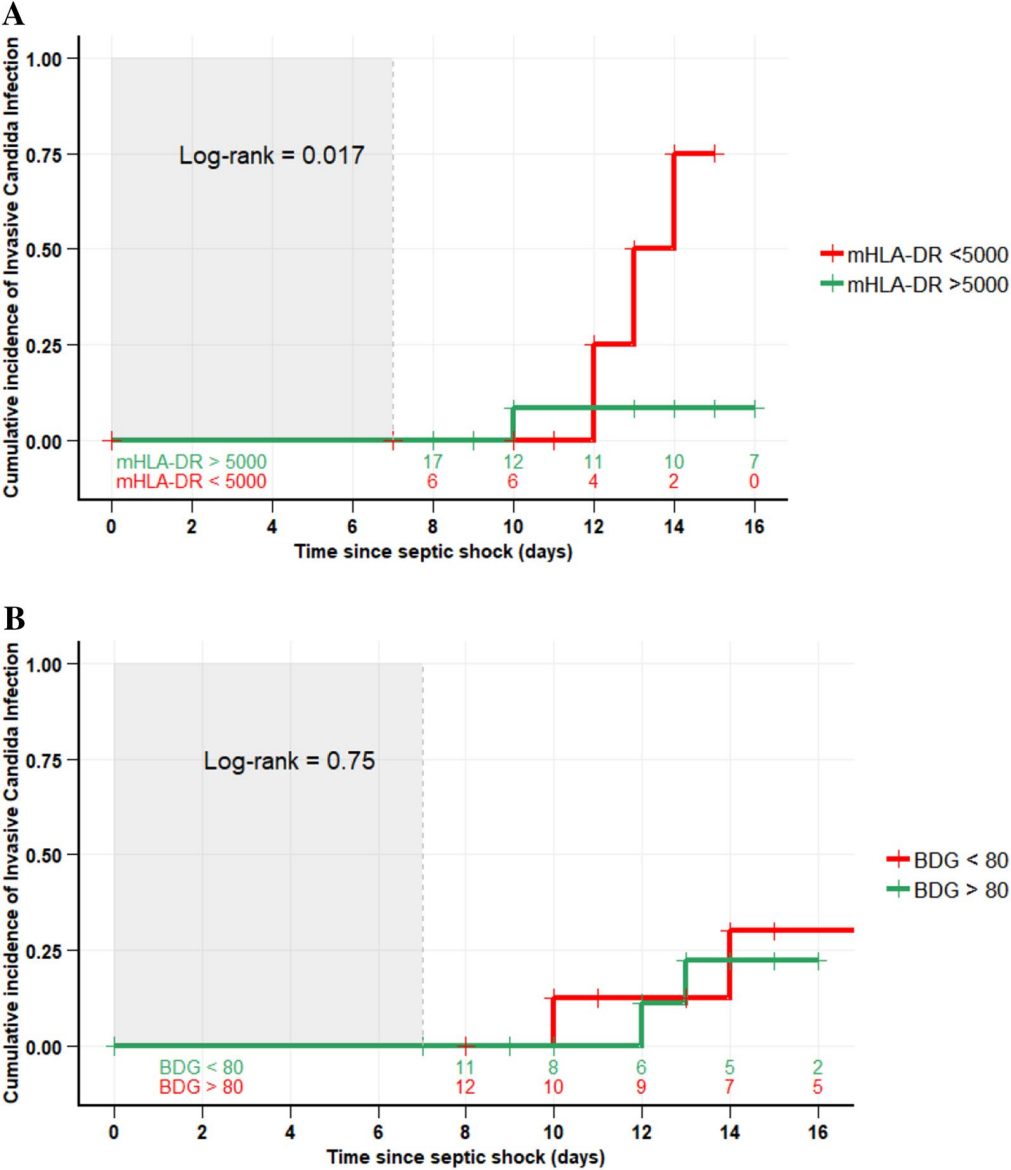
**A** Probability of Invasive Candida Infection after day 7 according to Day 7 mHLA-DR (Ab/cell). **B**. Probability of Invasive Candida Infection after day 7 according to day 7 β-d-glucan (pg/mL)

No other time-dependent immunological marker was associated with an increased risk of IC or death. Mycological markers were not significantly associated with IC; BDG value > 80 pg/mL have cs-HR 0.63 [0.14–2.86] for IC and 2.07 [0.4–10.77] for death. History of cancer was associated with IC (cs-HR 7.17 [1.38–37.36]) without increased risk of death (cs-HR 1.38 [0.25–7.61]). Results for sensitivity, specificity, PPV and NPV are shown in Table 3. The combination of mHLA-DR < 5000 Ab/c and BDG > 80 pg/mL provides a PPV of 33.3% [95% CI 6–60] and NPV of 84.2% [95% CI 72–95]. Patients with mHLA-DR < 5000 Ab/c and > 5000 Ab/c at day 7 had a significantly different cumulative incidence of IC after day 7 with a log-rank test of 0.017 (Fig. 3A). No difference was found between patients with BDG > 80 and < 80 pg/mL (Fig. 3B). A composite score combining mHLA-DR and BDG was calculated with a logistic regression model. Based upon a Monte Carlo simulation of 10,000 patients the potentially better ROC curve was next realized. Area under the ROC curve was 0.65.

**Table 3:** Predictive value of fungal and immunological markers

	Sensitivity (95% CI)		Specificity (95% CI)		PPV (95% CI)		NPV (95% CI)	
Past history of cancer	70	[41–98]	82.5	[70–94]	50.0	[23–76]	91.7	[82–100]
BDG > 80	70	[41–98]	42.5	[27–57]	23.3	[8–38]	85.0	[69–100]
mHLA-DR < 5000	60	[29–90]	57.5	[42–72]	26.1	[8–44]	85.2	[71–98]
Total lymphocytes < 500	80	[55–100]	45	[29–60]	26.7	[10–42]	90.0	[76–100]
CD4+T cells < 200	80	[55–100]	60	[44–75]	33.3	[14–52]	92.3	[82–100]
Association of markers								
mHLA-DR < 5000 and BDG > 80	40	[9–70]	80	[67–92]	33.3	[6–60]	84.2	[72–95]
Total lymphocytes < 500 and BDG > 80	70	[41–98]	62.5	[47–77]	31.8	[12–51]	89.3	[77–100]
CD4+T cells < 200/mm^3^ and BDG > 80	70	[41–98]	70	[55–84]	36.8	[15–58]	90.3	[79–100]
Past history of cancer and mHLA-DR < 5000	50	[19–81]	90	[80–99]	55.6	[23–88]	87.8	[77–97]

mHLA-DR: monocyte human leukocyte antigen-DR (AB/c); BDG: β-d-glucan (pg/mL); PPV: positive predictive value; NVP: negative predictive value. Lymphocytes, CD4+ T cell, CD8+ T cell (/mm^3^)

## Discussion

This prospective observational pilot study reports that a low value of mHLA-DR (< 5000 Ab/c) after septic shock is associated with a higher risk of invasive Candida infection. Adding mycological markers such as BDG do not improve the predictive value in a high-risk population.

Both innate and adaptive immune response is of importance for controlling Candida colonization and infection. Blood monocytes and tissue macrophages recognize pathogen-associated molecular patterns like fungal cell wall component. Antigen-presentation and subsequent cytokine secretion lead to T-helper lymphocyte activation and secondary adaptive immune response [27]. The expression of cell surface marker such as monocyte human leukocyte antigen-DR is regulated by multiple pro and anti-inflammatory mediators [4] and mHLA-DR level of expression reflects activation of monocytes and immune function. Thereby, low mHLA-DR expression has been described as a robust marker of immune dysfunction [28] and outcome following septic shock [4, 29, 30]. Furthermore, the expression of mHLA-DR lower than 8000 AB/c has been described to increase the risk of hospital acquired infection following septic shock or multiple trauma in the critically ill [21, 22, 31, 32]. In this study, mHLA-DR value < 8000 Ab/c was neither associated with IC nor death. Monocytic HLA-DR value < 5000 Ab/c was yet associated with IC. Our population is mainly patients with sepsis related to peritonitis. Leijte et al. found median mHLA-DR over 6000 Ab/c in bacterial peritonitis [33]. Invasive candida infection commonly appears in highly immunosuppressed patients and lower value of mHLA-DR could be associated with higher risk of IC. Continuing or declining immune deficiency is associated with an increased mortality [33]. Similarly, our results suggest an increased probability of IC when mHLA-DR remains < 5000 Ab/c after day 7 of septic shock.

We further explored whether Candida colonization and a fungal biomarker, BDG, could be of interest. Colonization and prediction scores have been developed to identify ICU patients at high risk of IC. In our study, colonization and Candida Score (≥ 3) were positive in all IC patients and 80% of NIC patients, consistent with the low positive predictive value reported elsewhere [23, 34–36].

Colonization-driven antifungal treatment exposes to unnecessary antifungal treatment and its consequence on antifungal resistances and ecology [37–39]. In this regard, fungal biomarkers such as BDG have received increasing attention. Recent studies have evaluated the BDG performance to predict IC and have reported high sensitivity and specificity [40, 41]. Others have reported mitigated results with sensitivity ranging from 40 to 100% [42–45]. Because of studies reporting high negative predictive value, BDG has been used either to rule out IC or to discontinue antifungal therapy as a part of antifungal stewardship [46–48]. In our study, the low negative predictive value and specificity of BDG suggests that BDG could not be used to rule IC out (Table 2). Furthermore, BDG could not be used to predict IC in the present study which is accordance with the recent study by Angebault et al. who reported a BDG sensitivity of 64% in candidemia [49]. This is also in line with studies that have shown that BDG driven therapy was not associated with outcome improvement [50, 51].

When the combination of both host response (mHLA-DR) and fungal biomarker (BDG) was evaluated, the positive and negative value was not improved compared to mHLA-DR alone. Indeed, BDG predictive value may be decreased in high-risk population (Candida score 3 in 84% of patients). Past history of cancer was associated with an increased risk of IC. This result was previously described by Lortholary [52]. An increased expression of negative T-cell co-stimulatory molecule (PD1, CTLA4) was described in both cancer [53] and candida-related sepsis [54] revealing possible immunological similarities.

The present study has some major limits. First, this is a single institution study with a relatively small cohort and a limited number of patients at day 7 because of deaths or discharge from the ICU. Second, 27% of patients in NIC group have received pre-emptive treatment at inclusion or during the ICU stay limiting IC occurrence in some of them. Third, we could have also evaluated other fungal biomarkers such as mannan/antimannan, *Candida albicans* germ tube antibody or T2Candida Panel that have been associated with a high negative predictive value, but we lacked funding and did choose to focus on a pair of pathogen and host-related biomarkers.

## Conclusion

This study suggests that a sepsis-induced immune dysfunction (mHLA-DR < 5000 Ab/c) is associated with a higher risk of ICU acquired invasive candida infection in patient with septic shock. Combination with fungal biomarker (BDG) did not enhance the prediction value. Larger studies are needed before implementing these results in the routine care.

## Data Availability

The datasets used and analyzed during the current study are available from the corresponding author on reasonable request.

## Appendix 1

**Table:**

Candida species	Colonization at inclusion (*n* = 34/50)	Colonization anytime (*n* = 41/50)	Invasive infection (*n* = 10/50)
Colonization/infection			
With only one specie	22 (65)	25 (60)	
With multiple species	12 (35)	14 (34)	
*Candida albicans*	20 (59)	23 (56)	7 (70)
*Candida glabrata*	9 (26)	10 (24)	1 (10)
*Candida parapsilosis*	9 (26)	10 (24)	1 (10)
*Candida tropicalis*	4 (12)	4 (10)	1 (10)
Other species	10 (29)	12 (29)	

## Abbreviations

BDG: β-d-Glucan
csHR: Cause-specific hazard ratio
IC: Invasive Candida infection
mHLA-DR: Monocyte human leukocyte antigen-DR
NIC: No invasive Candida infection
SAPSII: Simplified Acute Physiology Score II
SOFA: Sepsis-related Organ Failure Assessment
